# Extremely Low-Frequency Electromagnetic Fields Cause G1 Phase Arrest through the Activation of the ATM-Chk2-p21 Pathway

**DOI:** 10.1371/journal.pone.0104732

**Published:** 2014-08-11

**Authors:** Chao-Ying Huang, Cheng-Wei Chang, Chaang-Ray Chen, Chun-Yu Chuang, Chi-Shiun Chiang, Wun-Yi Shu, Tai-Ching Fan, Ian C. Hsu

**Affiliations:** 1 Department of Biomedical Engineering and Environmental Sciences, National Tsing Hua University, Hsinchu, Taiwan; 2 Institute of Statistics, National Tsing Hua University, Hsinchu, Taiwan; 3 Magnet Group, Instrumentation Development Division, National Synchrotron Radiation Research Center, Hsinchu, Taiwan; II Università di Napoli, Italy

## Abstract

In daily life, humans are exposed to the extremely low-frequency electromagnetic fields (ELF-EMFs) generated by electric appliances, and public concern is increasing regarding the biological effects of such exposure. Numerous studies have yielded inconsistent results regarding the biological effects of ELF-EMF exposure. Here we show that ELF-EMFs activate the ATM-Chk2-p21 pathway in HaCaT cells, inhibiting cell proliferation. To present well-founded results, we comprehensively evaluated the biological effects of ELF-EMFs at the transcriptional, protein, and cellular levels. Human HaCaT cells from an immortalized epidermal keratinocyte cell line were exposed to a 1.5 mT, 60 Hz ELF-EMF for 144 h. The ELF-EMF could cause G1 arrest and decrease colony formation. Protein expression experiments revealed that ELF-EMFs induced the activation of the ATM/Chk2 signaling cascades. In addition, the p21 protein, a regulator of cell cycle progression at G1 and G2/M, exhibited a higher level of expression in exposed HaCaT cells compared with the expression of sham-exposed cells. The ELF-EMF-induced G1 arrest was diminished when the *CHK2* gene expression (which encodes checkpoint kinase 2; Chk2) was suppressed by specific small interfering RNA (siRNA). These findings indicate that ELF-EMFs activate the ATM-Chk2-p21 pathway in HaCaT cells, resulting in cell cycle arrest at the G1 phase. Based on the precise control of the ELF-EMF exposure and rigorous sham-exposure experiments, all transcriptional, protein, and cellular level experiments consistently supported the conclusion. This is the first study to confirm that a specific pathway is triggered by ELF-EMF exposure.

## Introduction

The rising prevalence of electric appliances has increasingly exposed people to extremely low-frequency electromagnetic fields (ELF-EMFs). Over the past 30 years, both the mass media and the scientific community have expressed concerns about the biological effects of ELF-EMFs. The International Agency for Research on Cancer (IARC) has classified ELF-EMFs as a class 2B carcinogen, possibly carcinogenic to humans, based on epidemiological studies on childhood leukemia (IARC 2002) [1]. The biological mechanism facilitating the effects of ELF-EMFs remains unclear, but ELF-EMFs have been reported to induce various biological effects. Previous microarray studies have indicated that ELF-EMFs cause the differential expression of genes involved in metabolism, cellular physiological processes, signal transduction, and immune responses [2], [3]; however, other studies have reported that no significant difference in gene expression profiles was observed after ELF-EMF exposure [4]–[7]. Reports have stated that ELF-EMFs can promote cell proliferation [8]–[12], whereas others have indicated that ELF-EMF exposure inhibits cell proliferation [13]–[16]. Thus far, the potential hazards of ELF-EMF exposure remain unclear and convincing evidence and consistent results are lacking.

In this study, we investigated the biological effects of ELF-EMF exposure at the transcriptional, protein, and cellular levels. We set up the ELF-EMF exposure system shown in Figure S1. The effects of 1.5 mT, 60 Hz ELF-EMFs, on an immortalized epidermal keratinocyte cell line, human HaCaT keratinocyte [17] possessing 2 mutant p53 alleles [18], were evaluated. HaCaT cells are suitable experiment models for this study because epidermal keratinocyte cells are the cells first subjected to environmental stress in humans. We performed cDNA microarray experiments to screen differentially expressed genes (DEGs) of HaCaT cells after ELF-EMF exposure, followed by real-time PCR to confirm our microarray data. Immunoblotting were used to study the expression of proteins involved in the regulation of cell growth, after ELF-EMF exposure. We further applied flow cytometry to investigate the cell cycle of HaCaT cells distribution after ELF-EMF exposure. The ATM-Chk2-p21 pathway is identified for effects of the ELF-EMF exposure in HaCaT cells and confirmed by *CHK2* gene knockdown experiments. We conclude that ELF-EMFs activate the ATM-Chk2-p21 pathway in HaCaT cells, inhibiting cell proliferation.

## Materials and Methods

### Cell culture

Immortalized nontumorigenic human keratinocytes HaCaT [17] were kindly provided by Dr. N. E. Fusening (German Cancer Research Center, Heidelberg, Germany). HaCaT cells were cultured in Dulbecco’s Modified Eagle’s Medium (Gibco BRL, Grand Island, NY) supplemented with 10% heat-inactivated fetal bovine serum (HyClone, Laboratories Inc., Logan, UT) and antibiotics (100 U/ml of penicillin, 100 µg/ml of streptomycin, and 2 mM L-glutamine; Gibco BRL, Grand Island, NY) at 37°C in humidified air containing 5% CO_2_. HaCaT cells were passaged every 4 d upon 80%–90% confluence to maintain cells at the exponential phase. Both the sham-exposed (unexposed) and exposed cells used for each experiment were from the same culture flasks.

### ELF-EMF exposure system and UVB irradiation

A uniform 1.5 mT, 60 Hz ELF-EMF was generated using a Helmholtz coil system consisting of 100 turns of copper wire in each coil, and situated inside a tissue culture incubator. The pair of coil apparatuses each had a diameter of 34 cm and were 17 cm apart. AC power to the Helmholtz coil system was supplied by a step-down transformer connected to a 60-Hz, 110 V AC source. The background magnetic fields in the incubator were 0.47±0.03 µT (upper part), 0.25±0.01 µT (middle part), and 1.15±0.47 µT (lower part). The spatial variation of the background is due to the electronic devices of the incubator (NuAire, Inc. Plymouth, MN) attached at the upper part of it. We have also added a heater in the water pan of the incubator with feedback control and a water-cooling system to aid the original temperature control system of the incubator to ensure the temperature uniformity inside the incubator. Cells were exposed to an ELF-EMF in the central area of the two coils with a 1.5 mT (the spatial variation of the magnetic flux density was 4.4% in the exposure area), 60 Hz magnetic field. The magnetic field was measured using an EFA-3 field analyzer (Wandel & Goltermann, Eningen, Germany) with a 3 cm diameter probe. All sham-exposed cells were cultured in a chamber magnetically shielded using a mu-metal (AirCraftMaterialsUK, UK) box, in the same incubator as the exposed cells. There are 116 holes with a diameter of 1 cm on the mu-metal box to ensure the air circulation inside the whole space of the incubator. Our ELF-EMF exposure system is presented in Figure S1. When the coil system generated the uniform 1.5 mT ELF-EMF (the exposed environment), the intensity of ELF-EMF within the mu-metal box was 1.50±0.03 µT (the unexposed environment). The temperature in the incubator was monitored using a thermometer (TES, Taipei, Taiwan) in the unexposed (36.9±0.3°C) and exposed (36.9±0.3°C) environments. The pH value of the culture medium was measured using a Corning pH meter 320 (Corning, NY) and the pH value was 7.33±0.02 for the unexposed and 7.34±0.02 for the exposed environments.

The UVB-light source was a 6-W UVB fluorescent tube (model Spectroline EB-160C, Spectronics Corporation, Westbury, NY). The UVB dose was measured using a photometer (model IL 1400A, International Light Inc., Newburyport, MA). The positive control was collected 8 h after 233 J/m^2^ UVB irradiation. The UVB irradiation procedure has been described in detail in our previous studies [19], [20].

### Microarray experiment and data analysis

Most cells used for microarray in this study were viable at the time of harvest; nonviable (floating) cells were removed before harvesting for RNA extraction. Total RNA was isolated using TRIzol reagent (Invitrogen, Carlsbad, CA), and purified with an RNeasy Mini Kit (Qiagen, Valencia, CA). The quality of the total RNA was verified using an Agilent 2100 bioanalyzer with an RNA 6000 Nano Chip kit (Agilent Technologies, Palo Alto, CA). SuperScript II Reverse Transcriptase (Invitrogen, Carlsbad, CA) was used to perform RNA reverse transcription. cDNA was purified using a Microcon YM-30 column (Millipore, Billerica, MA) and labeled using a 3DNA Array 50™ kit (Genisphere, Hatfield, PA) following the manufacturer’s protocol. Hybridization was performed at 65°C in a water bath for 16 to 20 h. The arrays were washed according to the manufacturer’s protocol and scanned using a GenePix 4100A scanner (Axon Instruments, Union City, CA).

A total of 10 RNA samples were hybridized with 20 microarrays in a loop-design (Figure S2). Further details regarding the fabrication of cDNA microarrays and loop-design statistics are available in previous reports [21]–[23].

At the beginning of exposure experiments, all exposure samples were placed inside the mu-metal box with sham-exposed samples C1 and C2; the exposure samples were then moved to the exposure area at 96 h, 72 h, 48 h, 12 h, 8 h, and 4 h before the common end time point, respectively. Then all exposure samples as well as C1 and C2 were harvested at the common end time point. In this arrangement, all exposure samples can share the same set of sham control samples C1 and C2 (Figure S2).

As shown in Figure S2, C1 and C2 denote the 2 identical sham controls that were also used as the internal controls to evaluate any possible bias in the microarray system during the experimental process as well as data analysis. We conducted microarray data processing using 2 steps as follows: First, the differentially expressed genes (DEGs) were identified using an F test at a Bonferroni-adjusted significance level of 0.05 divided by the number of genes to be tested. Second, DEGs were combined with at least a 1.3-fold change denoted as selected genes (SGs). In this study, 1.3 was selected as the cut-off value because no DEGs existed between 2 identical sham controls (C1 and C2; internal control) when this criterion was used. The microarray data of this study were submitted to Gene Expression Omnibus (GEO, Series accession number GSE 45631).

### Quantitative real-time PCR

The microarray gene expression data of six cell cycle-related SGs were selected to be confirmed by quantitative real-time PCR (qRT-PCR). Total RNA was extracted using TRIzol reagent (Invitrogen, Carlsbad, CA), and purified with an RNeasy Mini Kit (Qiagen, Valencia, CA). Six µg of purified RNA were reverse transcribed to cDNA using a High Capacity cDNA Reverse Transcription Kit (Applied Biosystems, Foster City, CA). Subsequently, 100 ng of cDNA was used to carry out qRT-PCR amplification with the 2X Power SYBR Green PCR Master Mix (Applied Biosystems, Warrington, UK) on an ABI 7300 sequence detector (Applied Biosystems, Foster City, CA). The primer sequences of the genes were: *GAPDH* forward 5′-GGG GAG CCA AAA GGG TCA T-3′ and reverse 5′-CCA GGG GTG CTA AGC AGT TG-3′; *CDKN1A* (encodes p21 protein) forward 5′-TTC TAC CAC TCC AAA CGC CG-3′ and reverse 5′-GCA GAA GAT GTA GAG CGG GC-3′; *CDC25B* forward 5′-CTG CCA TGT TGC CCC TTT CT-3′ and reverse 5′-GAA GTG TCC TGA GAT GGG CGT-3′; *CDC20* forward 5′-GAC CGC TAT ATC CCC CAT CG-3′ and reverse 5′-TTC CTT CTT GGT GGG CGT CT-3′; *CDC2* forward AGG ATT TTC AGA GCT TTG GGC-3′ and reverse 5′-ATG CTA GGC TTC CTG GTT TCC-3′; *CCNA2* forward 5′-GCA CTG GTG GTC TGT GTT CTG T-3′ and reverse 5′-CTT CTT GGA TGC CAG TCT TAC TCA-3′; *CCNB1* forward 5′-TGA CAT GGT GCA CTT TCC TCC-3′ and reverse 5′-AGG TGC TGC ATA ACT GGA AGA AG-3′.

The PCR reaction was operated using the following conditions: 40 cycles of denaturing (95°C, 15 s), annealing (60°C, 30 s), and extension (72°C, 45 s) processes. The relative expression level of mRNAs was normalized to that of *GAPDH* (used as a reference gene in real-time PCR experiments) in each sample. All experiments were performed in triplicates to confirm reproducibility.

### Cell growth curve

HaCaT cells (1.0–1.4×10^5^) were seeded in a 10 cm tissue culture dish (Orange Scientific, Braine-l'Alleud, Belgium) and incubated for 12 h prior to exposure to an ELF-EMF. HaCaT cells were exposed to a 1.5 mT, 60 Hz field in the Helmholtz coil system for the whole periods of the experiments (24, 48, 72, 96, 120, and 144 h). The sham-exposed cells were cultured in the same incubator but shielded by mu-metal. At each time point in the experiments, inviable cells were determined and excluded using trypan blue (Sigma, St. Louis, MO) dye exclusion counting using a hemocytometer. All experiments were performed in triplicate to confirm reproducibility.

### Colony formation assay

The procedures for the colony assay of adherent cells were performed as described in previous articles [24]–[27]. In brief, both sham and exposed cells were seeded in 10 cm tissue culture dishes (Orange Scientific, Braine-l'Alleud, Belgium), 500 cells per dish, containing 15 ml of DMEM. Cells were allowed to attach to the Petri dishes for 12 h before ELF-EMF exposure and the attachment of cells was verified using a microscope. After 144 h of ELF-EMF exposure at 37°C, all the exposed cells were transferred from the exposure area to the magnetic shielded mu-metal box in the same incubator for 8 d. During clonal expansion, both the sham and exposed cells were not disturbed until the day for harvesting. The colonies of each petri dish were washed with PBS and fixed with 3 ml of Carnoy’s solution (methanol:acetic acid 3∶1, v/v) for 3 min. Then cells were washed with PBS and fixed in 100% methanol for 30 min followed by staining with KaryoMAX Giemsa Stain Solution (Gibco BRL, Grand Island, NY) for 10 min. Each dish was washed with water and dried overnight at room temperature. A colony is defined as a cluster of more than 50 cells. To perform the statistical significance tests we conducted colony formation assay in triplicates.

### Cell cycle analysis using propidium iodide (PI) staining

HaCaT cells were harvested and fixed with 6 ml of prechilled 83% ethanol/PBS at −20°C overnight. The fixed cells were centrifuged at 1500 rpm for 3 min and washed twice with ice-cold PBS. The resulting pellet was resuspended in 20 µg/ml of propidium iodide (Molecular Probes Inc., Eugene, OR) solution with 200 µg/ml of RNase A (Invitrogen, Carlsbad, CA) and 0.02% Triton X-100 (Sigma, St. Louis, MO), then incubated in the dark at 37°C for 30 min followed by FACSCanto flow cytometry (Becton Dickinson, San Jose, CA) to perform DNA content analysis. Data were acquired by collecting signals using channel PE-A. Each cell cycle phase was analyzed using ModFit LT software (Verity Software House, Topsham, ME).

### Immunoblotting assay

After exposure to an ELF-EMF, cells were washed with ice-cold PBS and lysed using RIPA buffer (Cell Signaling Technology, Beverly, MA) supplemented with the Protease Inhibitor Cocktail, Phosphatase Inhibitor Cocktail 2, and Phosphatase Inhibitor Cocktail 3 (Sigma-Aldrich, St. Louis, MO) according to the manufacturer’s protocol. Protein concentrations were determined using a Bicinchoninic Acid Protein Assay Kit (Sigma-Aldrich, St. Louis, MO). The equal amounts of total protein were separated using SDS-PAGE, transferred onto a nitrocellulose membrane (Pall, Pensacola, FL) for western blotting. Membranes were blocked with 5% (w/v) nonfat dry milk in TBST buffer (Cell Signaling Technology, Beverly, MA) for 1 h at room temperature. After blocking, membranes were incubated with primary antibodies overnight and gentle agitation at 4°C followed by incubation in horseradish peroxidase conjugated secondary antibodies for 1 h at room temperature. The protein bands were developed using SuperSignal West Femto Maximum Sensitivity Substrate (34096; Pierce Biotechnology, Thermo Scientific, Rockford, IL) and photographed in a G:Box Chemi XT16 system (Syngene, Frederick, MD).

The primary antibodies used for western blotting were: ATM (2873), Chk2 (3440), phospho-Chk2 Thr68 (2661), and p21 (2946) from Cell Signaling Technology (Beverly, MA); phospho-ATM Ser1981 (2152-1) from Epitomics (Burlingame, CA); and β-Actin (sc-47778) from Santa Cruz (Santa Cruz, CA). The horseradish peroxidase conjugated secondary antibodies were: anti-mouse IgG HRP-linked antibody (7076) and anti-rabbit IgG HRP-linked antibody (7074) from Cell Signaling Technology (Beverly, MA).

### siRNA transfection

HaCaT cells (1.0×10^5^ cells) were seeded in a 10 cm tissue culture dish (Orange Scientific, Braine-l'Alleud, Belgium) and incubated for 16 h before siRNA transfection. The siRNA transfection was conducted using Lipofectamine RNAiMAX Reagent (Invitrogen, Carlsbad, CA) according to the manufacturer’s instructions. Cells were transfected with 5 nM of *CHK2* siRNA or scrambled siRNA and subsequently exposed to a 1.5 mT ELF-EMF and harvested at 96 to 144 h. Cells were retransfected with siRNA after 72 h to maintain siRNA knockdown efficiency for 120 and 144 h. Both of the siGENOME SMARTpool siRNA against *CHK2* (M-003256-06) and siGENOME Non-Targeting siRNA Pool #2 (D-001206-14) (used as scrambled siRNA) were purchased from Dharmacon (Lafayette, CO).

### Statistical analysis

All data are presented as mean ± SD and were obtained after conducting all experiments in triplicate and analyzed using Prism 5 software (GraphPad Software, San Diego, CA). Differences between the sham and exposed groups were analyzed using Student’s *t*-test and considered statistically significant at *P*<0.05.

## Results

### Microarray data revealed gene expression responses to ELF-EMF exposure in HaCaT cells

To screen differentially expressed genes (DEGs) of HaCaT cells after 4 to 96 h of ELF-EMF exposure, we conducted loop-designed cDNA microarray experiments [28] which was shown in Figure S2. Two identical sham-exposed samples (C1 and C2) were cultured inside the magnetic shielded mu-metal box, and the difference between the expressions of C1 and C2 was considered as an internal control of the system. The difference in expressions between the sham (the mean of the expressions of C1 and C2) and that of the exposed groups was defined as differentially expressed levels. The positive control was collected 8 h after 233 J/m^2^ UVB irradiation. During data processing, the DEGs were identified by applying the criteria of the Bonferroni-adjusted F test, *P*<0.05 for each null hypothesis, and then denoted as selected genes (SGs) in combination with at least a 1.3-fold change. Table S1 shows the number of DEGs and SGs in each exposed group, and no SG was observed between the internal control samples. The SGs of the positive control revealed genes involved in oxidative phosphorylation and ribosome pathways, which are in good agreement with prior studies in which other human cell types were used as experimental models [22], [29]–[32]. The results of the internal and positive controls indicated that the loop-designed microarray approach is a reliable system for screening the transcriptional profiles of HaCaT cells exposed to ELF-EMFs.

Exposure to ELF-EMF for 96 h resulted in 102 SGs, which are listed in Table S2. Six genes were grouped into cell cycle pathways according to the Kyoto Encyclopedia of Genes and Genomes (KEGG) [33]. One upregulated gene (*CDKN1A*) and 5 downregulated genes (*CDC25B*, *CDC20*, *CDC2*, *CCNA2*, *CCNB1*) were validated using a quantitative real-time PCR (qRT-PCR) (R^2^ = 0.90; Figure S3A). These gene expression results indicated that ELF-EMF exposure is possible to inhibit the cell cycle progress of HaCaT cells. The same 6 cell cycle-related SGs after 120 and 144 h of ELF-EMF exposure were further analyzed using qRT-PCR. The results showed that they were similarly regulated after 96, 120, and 144 h of ELF-EMF exposure (Figure S3B).

### Inhibition of HaCaT cell growth after ELF-EMFs exposure

**Figure 1:**
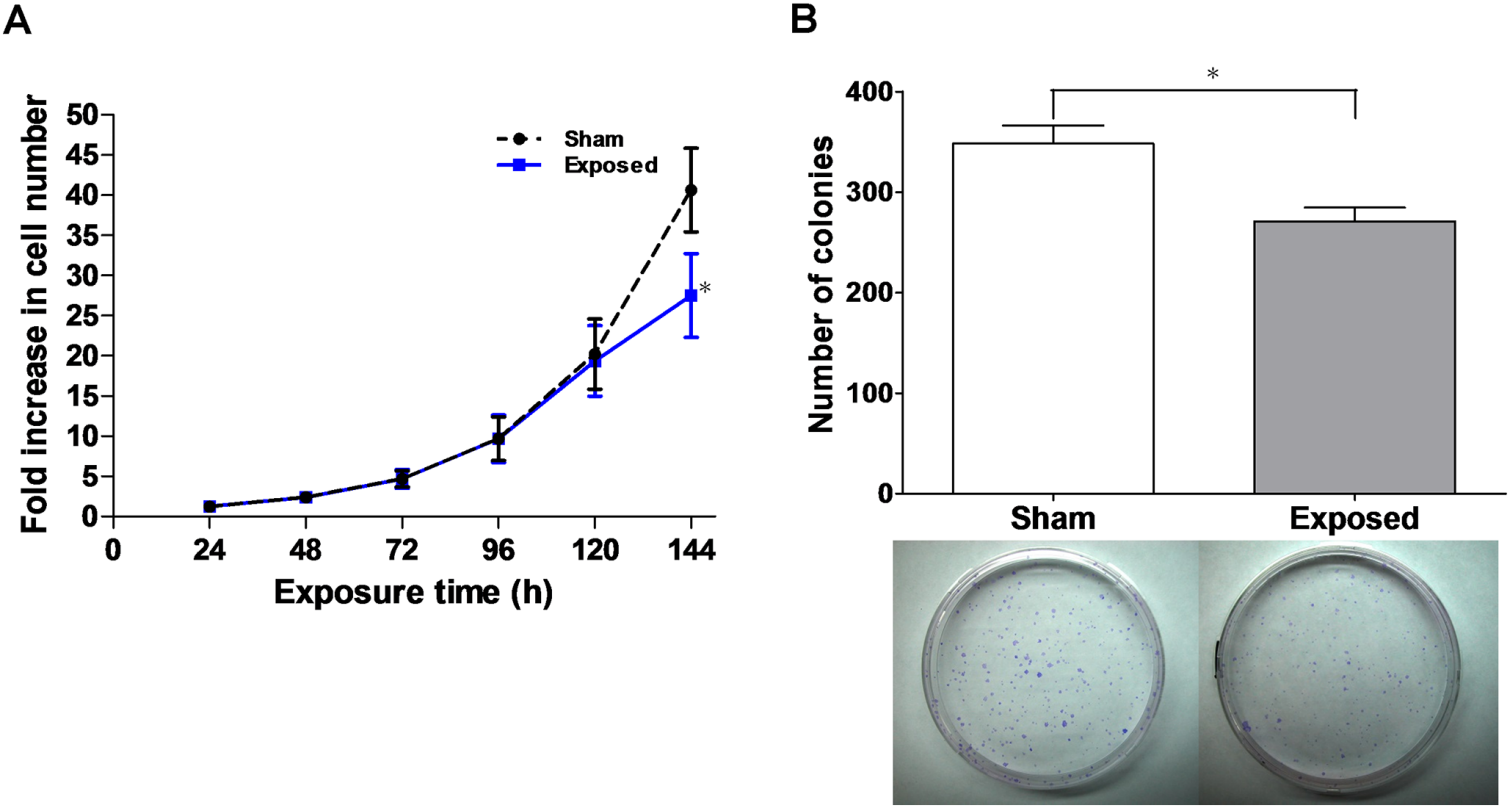
ELF-EMF inhibited cell growth and decreased cell colony formation. (A) Growth curves of HaCaT cells for the sham (•) and exposed (▪) groups. The HaCaT cells were seeded and incubated for 12 h prior to ELF-EMF exposure. The results were presented as the mean ± SD fold change in the cell count relative to the initial cell count of three independent experiments. (B) Colony formation assay. In the upper panel, the bars show the colony formation of the sham and exposed cells for 144 h of ELF-EMF exposure. The results are presented as the mean ± SD of three independent experiments. In the lower panel, they were representative for sham (left) and exposed (right). The Student’s *t*-test was used to analyze the differences between the sham and exposure groups. *P<0.05.

In our original cell growth study, no significant differences were observed between the sham and exposed groups after up to 96 h of ELF-EMF exposure. Inspired by the gene expression results of cell cycle-related SGs, we extended the exposure time to 144 h to further investigate the effects of ELF-EMFs on the growth of HaCaT cells. As shown in Figure 1A, sham-exposed cells grew exponentially at all the time points whereas the growth rate of the exposed cells significantly decreased (68.43%±8.46% of sham-exposed cells) at the 144-h time point. The data suggested that exposure to ELF-EMFs for 144 h inhibited HaCaT cell growth.

### ELF-EMFs decreased cell proliferation efficiency

In addition to cell growth, we performed colony formation assays on HaCaT cells to study cell proliferation efficiency after 144 h of ELF-EMF exposure. Figure 1B showed that the exposed cells presented a decreased (77.80%±3.88% of sham-exposed cells) colony production of HaCaT cells compared with that of the sham-exposed cells. These data indicated that ELF-EMFs decrease cell proliferation.

### G1-phase cell cycle arrest induced by ELF-EMFs

**Figure 2:**
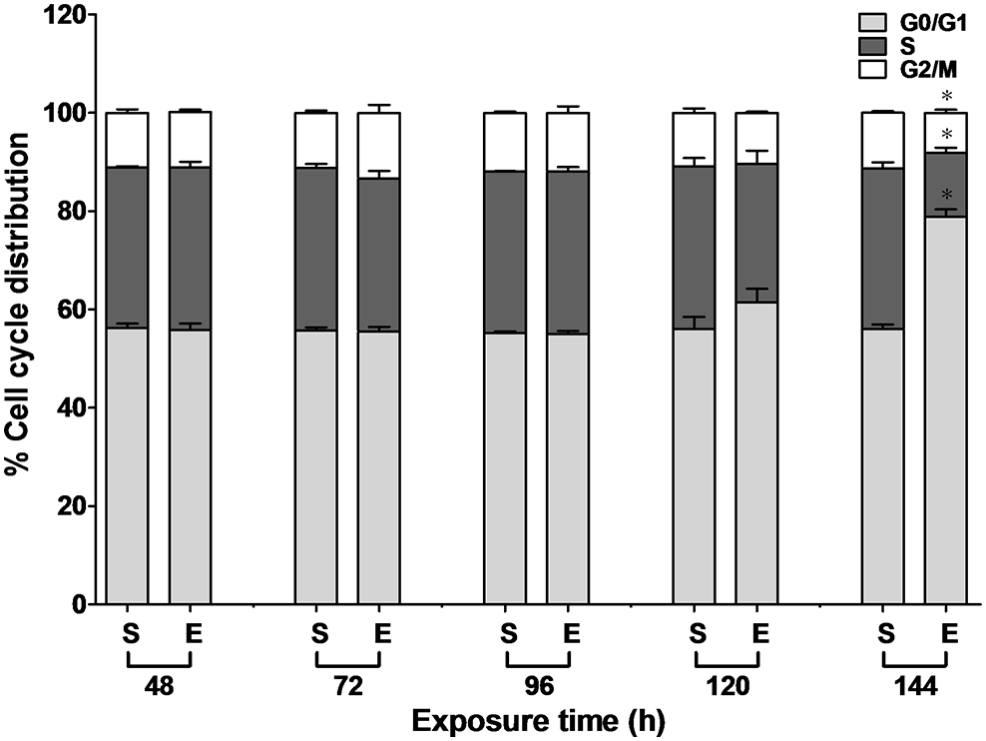
Cell cycle distribution in the sham (S) and exposed (E) HaCaT cells under ELF-EMF exposure. HaCaT cells were exposed to ELF-EMF for 48 to 144 h and analyzed by PI staining and flow cytometry. The percentage of the cell cycle growth phases were represented as the mean ± SD of three independent experiments. The Student’s *t*-test was used to analyze the differences between the sham and exposure groups. *P<0.05.

To investigate whether ELF-EMFs disturbed cell cycle progression, the cell cycle distribution between 48 and 144 h of ELF-EMF exposure was analyzed by propidium iodide (PI) staining and flow cytometry. As shown in Figure 2, at the 144-h time point, the percentage of exposed cells in the G0/G1 phase (78.82%±1.57%) was higher than that in the sham-exposed cells (56.02%±0.94%). No significant difference in the sub-G0/G1 phase of cell fractions was observed between the sham and exposed cells. This demonstrated that the G1-phase cell cycle arrest was induced after 144 h of ELF-EMF exposure.

### Activation of the ATM/Chk2-dependent signaling cascades responsible for ELF-EMF-induced inhibition of HaCaT cell growth

**Figure 3:**
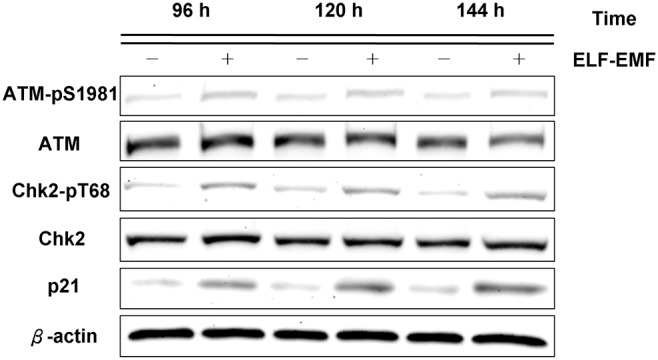
Immunoblotting of phospho-ATM (Ser1981), phospho-Chk2 (Thr68), p21 after ELF-EMF exposure in HaCaT cells. The expression levels of phospho-ATM (Ser1981), phospho-Chk2 (Thr68), and p21 in the exposed cells were higher than those in the sham-exposed cells at the indicated times. β-actin was used as a loading control. All proteins were determined in whole cell lysates from the sham and exposed HaCaT cells after the indicated exposure times.

Based on the cell cycle distribution results, we implemented immunoblotting assays to further investigate the expression of the proteins involved in the regulation of cell growth after ELF-EMF exposure. Figure 3 showed that the exposed HaCaT cells expressed higher levels of p21, a regulator of cell cycle progression at G1, than the sham-exposed cells did. These results suggested that ELF-EMF exposure inhibited cell cycle progression and that the G1 arrest was mediated by the induction of p21. Because HaCaT cells harbor both of the mutated alleles of p53 [18], the mutated p53 in HaCaT cells could not transcriptionally activate p21 [34], [35]. Therefore, the p21 in HaCaT cells was induced through a p53-independent pathway. Phospho-Chk2 (Thr68) is associated with the p21 in HaCaT cells rather than with p53 [36]. Figure 3 depicted the increase in the expression level of phospho-Chk2 (Thr68) in exposed cells. Because Chk2 is phosphorylated at Thr68 by phospho-ATM (Ser1981) [37], [38], phospho-ATM (Ser1981) did show a higher level of expression in the exposed cells than in the sham-exposed cells (Figure 3). These immunoblotting data suggested that HaCaT cell growth was possibly inhibited through the ATM-Chk2-p21 pathway.

**Figure 4:**
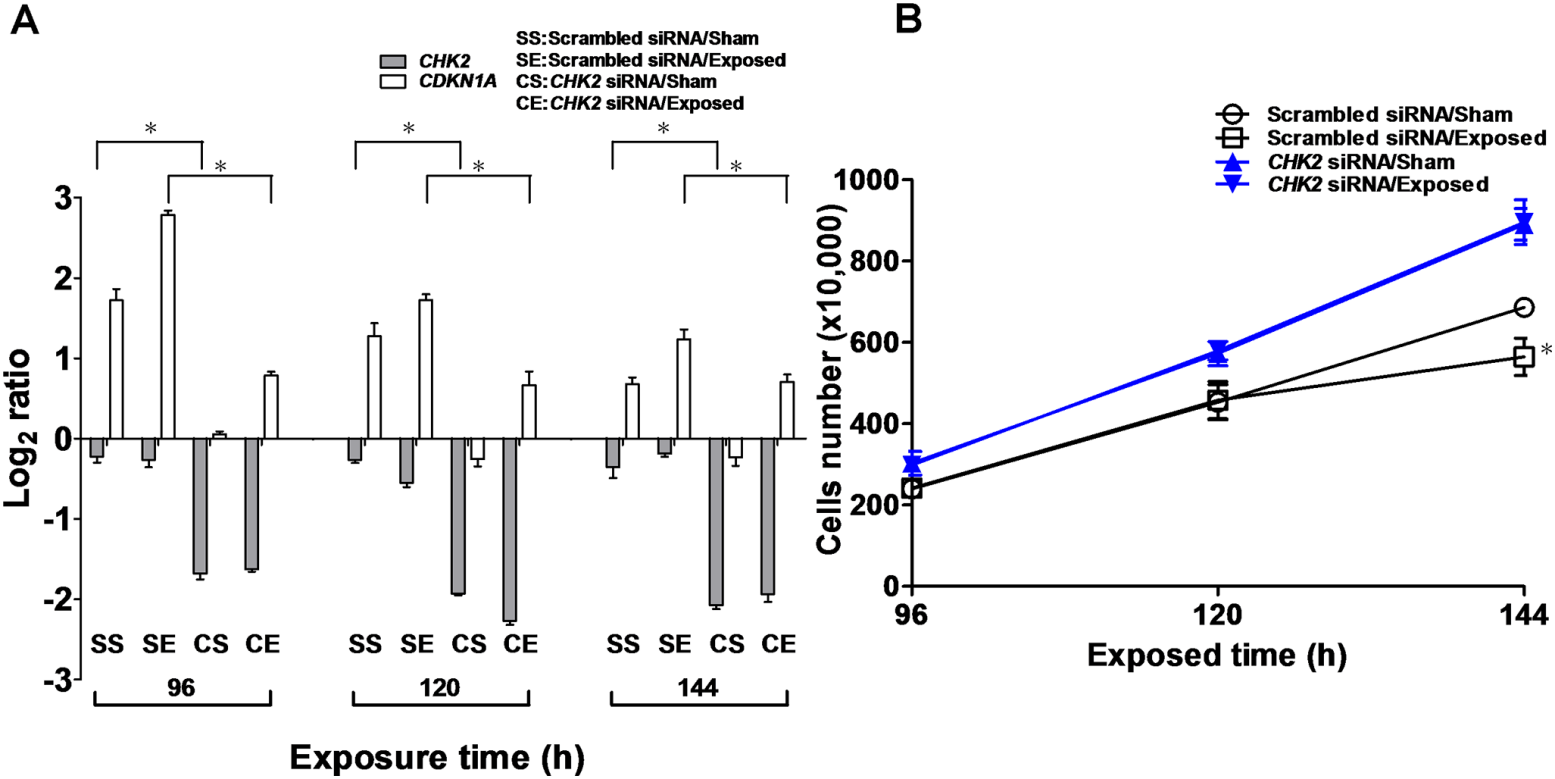
HaCaT cells were transfected with *CHK2* siRNA or scrambled siRNA, followed by exposure to ELF-EMFs. (a) Expression levels of *CHK2* and *CDKN1A* genes in the *CHK2* siRNA-treated and scrambled siRNA-treated group after 96 to 144 h of ELF-EMF exposure measured by qRT-PCR. (b) Growth curves of the *CHK2* siRNA-treated (solid) and scrambled siRNA-treated (hollow) HaCaT cells. The HaCaT cells (1.0×10^5^ cells) were seeded in a 10 cm tissue culture dish and incubated for 16 h prior to siRNA transfection. The results are presented as the mean ± SD of three independent experiments. The Student’s *t*-test was used to analyze the differences between the sham and exposure groups. *P<0.05.

To confirm that Chk2 contributes to the p53-independent upregulation of p21 when HaCaT cells were exposed to ELF-EMFs, specific small interfering RNA (siRNA) was applied to knock down the *CHK2* gene. By applying a Student’s *t*-test on the data (Figure 4A), a significantly decreased gene expression of *CHK2* was observed in the *CHK2* siRNA-treated cells compared with the scrambled siRNA-treated cells. The data also showed that the *CDKN1A* gene (which encodes the p21 protein) expression in the scrambled siRNA-treated cells was significantly higher than that in the *CHK2* siRNA-treated cells when exposed to ELF-EMF (Figure 4A). Whether *CHK2* gene knockdown affected the inhibition of cell growth induced by ELF-EMFs was then determined. As shown in Figure 4B, no significant difference between the cell growths of the sham and exposed cells treated with *CHK2* siRNA was observed. The results showed that the cell growth of the sham and exposed cells treated with scrambled siRNA was significantly different at the 144-h time point. We also investigated the cell cycle distribution of siRNA-treated HaCaT cells exposed to ELF-EMF (Figure S4). The cell cycle distribution data indicated that the *CHK2* siRNA-treated cells abrogated G1 arrest induced by ELF-EMF exposure. The siRNA results confirmed that ELF-EMFs activate the ATM-Chk2-p21 pathway in HaCaT cells, which results in cell cycle arrest at the G1 phase.

## Discussion

In the past 3 decades, in vitro studies on the biological effects of ELF-EMFs have been conducted using a variety of experimental designs, which involve varying intensities, durations of exposure, and cell types, have yielded inconsistent conclusions [39], [40]. Similar to most of the previous HaCaT cellular studies [9], [10], cells were exposed to ELF-EMFs for up to 96 h in this study. The cell growth and cell cycle results did not reveal a significant difference between the sham and exposed groups. However, microarray data indicated that the expression of six cell cycle-related genes was significantly differentiated after 96 h of ELF-EMF exposure. These significantly differentiated genes included one cyclin-dependent kinase (CDK) inhibitor gene (*CDKN1A*), one CDK gene (*CDC2*, known as *CDK1*), 2 cyclin genes (*CCNA2* and *CCNB1*), and 2 cell cycle division genes (*CDC25B* and *CDC20*). During cell cycle progression, to ensure that all cell cycle phases are executed in the correct order, the transition from one cell cycle phase to the next is controlled by the association of cyclin/CDK complexes (such as Cyclin A/CDK1 and Cyclin B/CDK1) [41], which can be activated by Cdc25 [42], [43]. Cdc20 is required to activate the anaphase-promoting complex (APC) for exit from mitosis [44], [45], whereas the overexpression of *CDKN1A* may result in both G1 and G2 arrest [46], [47]. These results implied that ELF-EMFs may cause cell cycle arrest after 96 h of ELF-EMF exposure. Based on the gene expression results, we revised the experiment design and extended the ELF-EMF exposure time to 144 h, which enabled the discovery of the new results presented in this study (i.e., cell growth revealed effects beyond 120 h of ELF-EMF exposure). Some studies have shown controversial results regarding the relationship between HaCaT cell proliferation and ELF-EMFs [9], [10], but their data presented nonexponential growth in sham-exposed cells.

In this study, to ensure that the sham-exposed groups were incubated in exactly the same environmental conditions as the exposed groups (with the exception of ELF-EMF exposure), we arranged the sham-exposed groups to be grown in the same incubator by using mu-metal to shield the ELF-EMFs. However, there is a shortage in this study due to the lack of a reliable positive control of ELF-EMF exposure. Nevertheless, the biological effects of ELF-EMFs were comprehensively evaluated at the transcriptional, protein, and cellular levels. It was concluded that ELF-EMFs can induce the ATM-Chk2-p21 pathway and trigger cell cycle arrest at the G1 phase in HaCaT cells. The identification and confirmation of the pathway assures our discovery of the ELF-EMF biological effects. The study findings can be used as a foundation for future studies regarding the biological effects of ELF-EMFs. Also based on this study, HaCaT cells can be used as a biological positive control for future study regarding the biological effects of ELF-EMFs.

## Supporting Information

Figure S1 **Photograph of 1.5 mT, 60 Hz ELF-EMF exposure system.** The ELF-EMF exposure system consists of (a) water-cooling system, (b) Helmoltz coil, (c) mu-metal magnetic shielding chamber, and (d) water pan.
(TIF)

Figure S2 **Schematic representation of loop-design microarray experiments.** Loop design of the microarray experiment for exposure to ELF-EMF. The symbols 4, 8, 12, 24, and 96 h denote the exposure times of samples, whereas C1 and C2 denote the sham exposure, which were also used as the internal controls of the system. The symbol UV represents a positive control harvested after 8 h 233 J/m^2^ UVB irradiation. Each arrow indicates a microarray hybridization experiment. The arrowheads and tails represent samples labeled with Cy5 and Cy3, respectively.
(TIF)

Figure S3 **Comparison of microarray data and qRT-PCR data of six cell cycle-related SGs after ELF-EMF exposure.** (a) Validation of the microarray data of six cell cycle-related SGs (*CDKN1A*, *CDC25B*, *CDC20*, *CDC2*, *CCNA2*, and *CCNB1*) after 96 h of ELF-EMF exposure in HaCaT cells by using qRT-PCR. *GAPDH* was used as a normalizer (reference gene). The qRT-PCR analysis of these genes verified the microarray data (R^2^ = 0.90). The x-axis represents the log_2_ ratio of the gene expression level of the microarray data, and the y-axis represents that of qRT-PCR data. The ratios indicate that gene expression levels measured from microarray and qRT-PCR were normalized to the respective gene expression levels of sham exposure. (b) The gene expression of the same 6 cell cycle-related SGs after 120 and 144 h of ELF-EMF exposure were analyzed using qRT-PCR. The qRT-PCR data are represented as the mean ± SD of three independent experiments.
(TIF)

Figure S4 **Cell cycle distribution in the sham (S) and exposed (E) of the** ***CHK2*** **siRNA-treated and scrambled siRNA-treated HaCaT cells under ELF-EMF exposure.** HaCaT cells were transfected with 5 nM of *CHK2* siRNA or scrambled siRNA, followed by exposure to 1.5 mT ELF-EMFs for 96 to 144 h. After exposure to ELF-EMFs, the *CHK2* siRNA-treated cells (right panel) and scrambled siRNA-treated cells (left panel) were analyzed by PI staining and flow cytometry. The percentage of the cell cycle growth phases are represented as the mean ± SD of three independent experiments. By applying a Student’s *t*-test on the data, there is no significant difference in cell cycle distribution between sham and exposed cells in *CHK2* siRNA-treated (right panel) and scrambled siRNA-treated (left panel) groups respectively.
(TIF)

Table S1 **Number of differentially expressed genes (DEGs), and selected-genes (SGs; DEGs with at least 1.3 fold change) in each ELF-EMF-exposed group.**
(DOC)

Table S2 **The list of 102 selected genes (SGs) after 96 h of ELF-EMF exposure.**
(DOC)
