# Supplementary figures and images for: Extremely Low-Frequency Electromagnetic Fields Cause G1 Phase Arrest through the Activation of the ATM-Chk2-p21 Pathway
Source: PLoS One. 2014 Aug 11;9(8):e104732. doi: 10.1371/journal.pone.0104732 (PMC4128733; doi:10.1371/journal.pone.0104732)

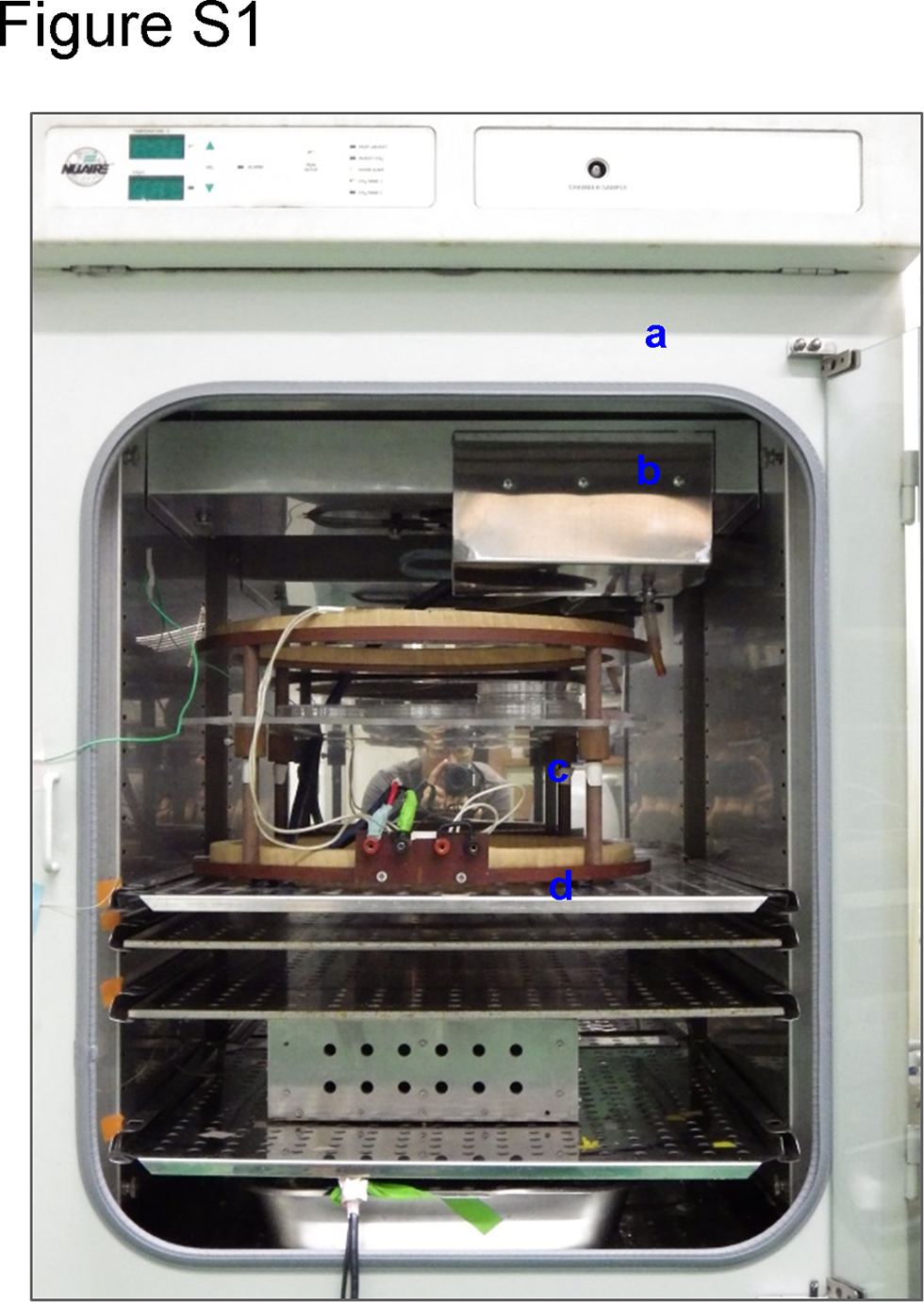

Supplement: Figure S1 — Photograph of 1.5 mT, 60 Hz ELF-EMF exposure system. The ELF-EMF exposure system consists of (a) water-cooling system, (b) Helmoltz coil, (c) mu-metal magnetic shielding chamber, and (d) water pan. (TIF) [file pone.0104732.s001.tif]

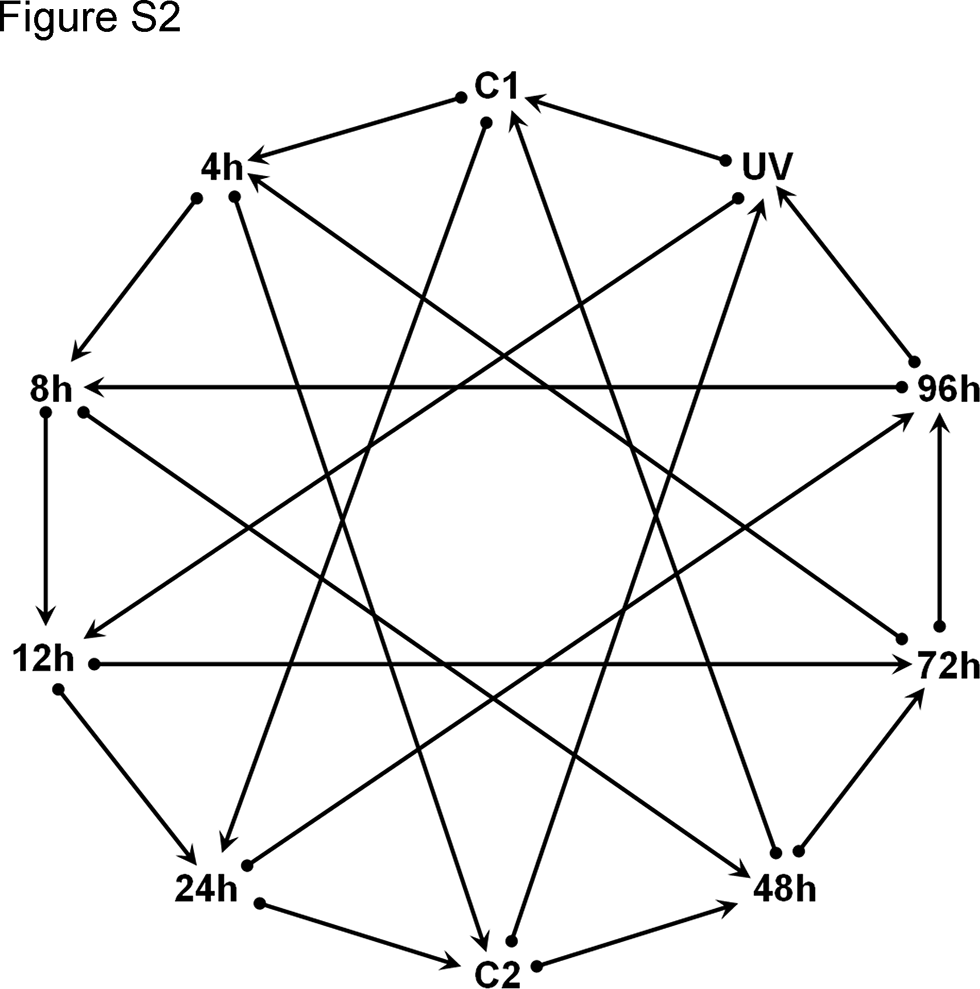

Supplement: Figure S2 — Schematic representation of loop-design microarray experiments. Loop design of the microarray experiment for exposure to ELF-EMF. The symbols 4, 8, 12, 24, and 96 h denote the exposure times of samples, whereas C1 and C2 denote the sham exposure, which were also used as the internal controls of the system. The symbol UV represents a positive control harvested after 8 h 233 J/m2 UVB irradiation. Each arrow indicates a microarray hybridization experiment. The arrowheads and tails represent samples labeled with Cy5 and Cy3, respectively. (TIF) [file pone.0104732.s002.tif]

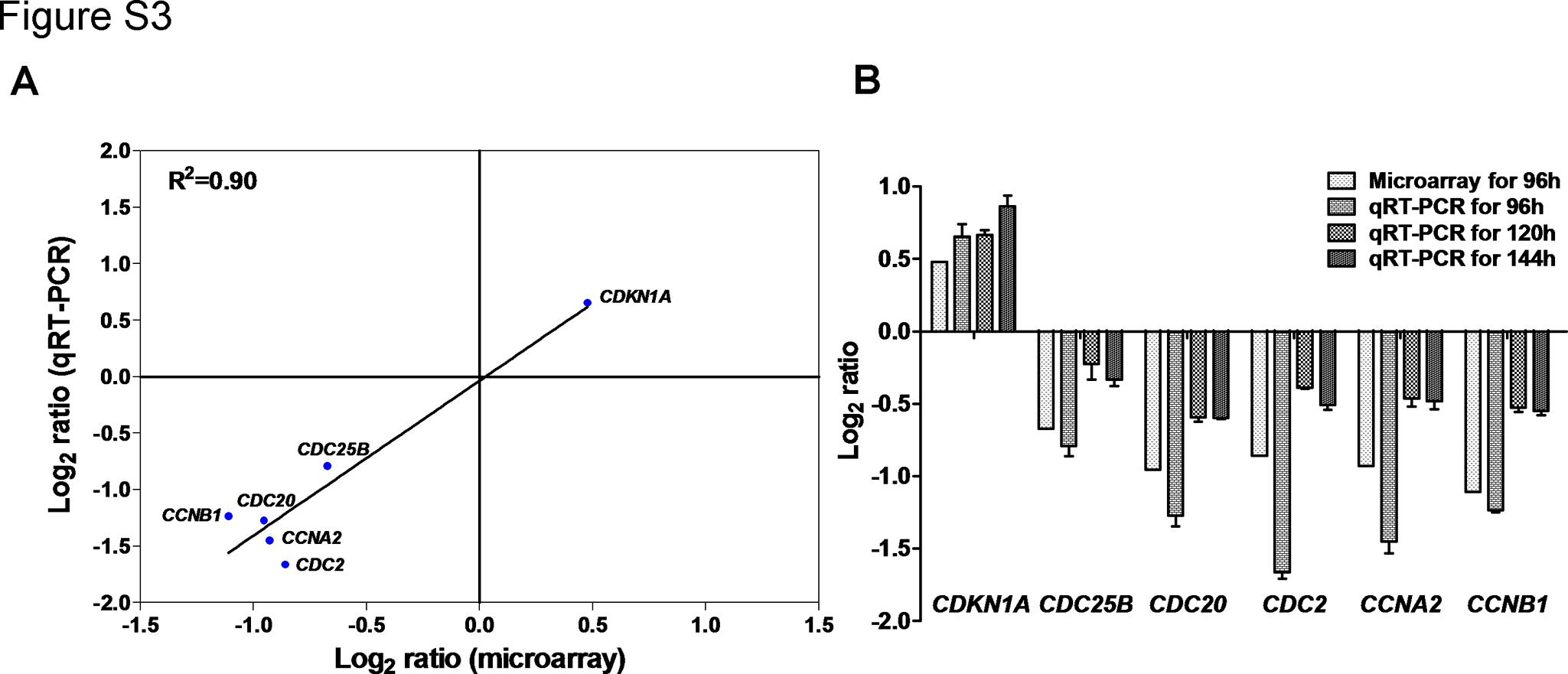

Supplement: Figure S3 — Comparison of microarray data and qRT-PCR data of six cell cycle-related SGs after ELF-EMF exposure. (a) Validation of the microarray data of six cell cycle-related SGs (CDKN1A, CDC25B, CDC20, CDC2, CCNA2, and CCNB1) after 96 h of ELF-EMF exposure in HaCaT cells by using qRT-PCR. GAPDH was used as a normalizer (reference gene). The qRT-PCR analysis of these genes verified the microarray data (R2 = 0.90). The x-axis represents the log2 ratio of the gene expression level of the microarray data, and the y-axis represents that of qRT-PCR data. The ratios indicate that gene expression levels measured from microarray and qRT-PCR were normalized to the respective gene expression levels of sham exposure. (b) The gene expression of the same 6 cell cycle-related SGs after 120 and 144 h of ELF-EMF exposure were analyzed using qRT-PCR. The qRT-PCR data are represented as the mean ± SD of three independent experiments. (TIF) [file pone.0104732.s003.tif]

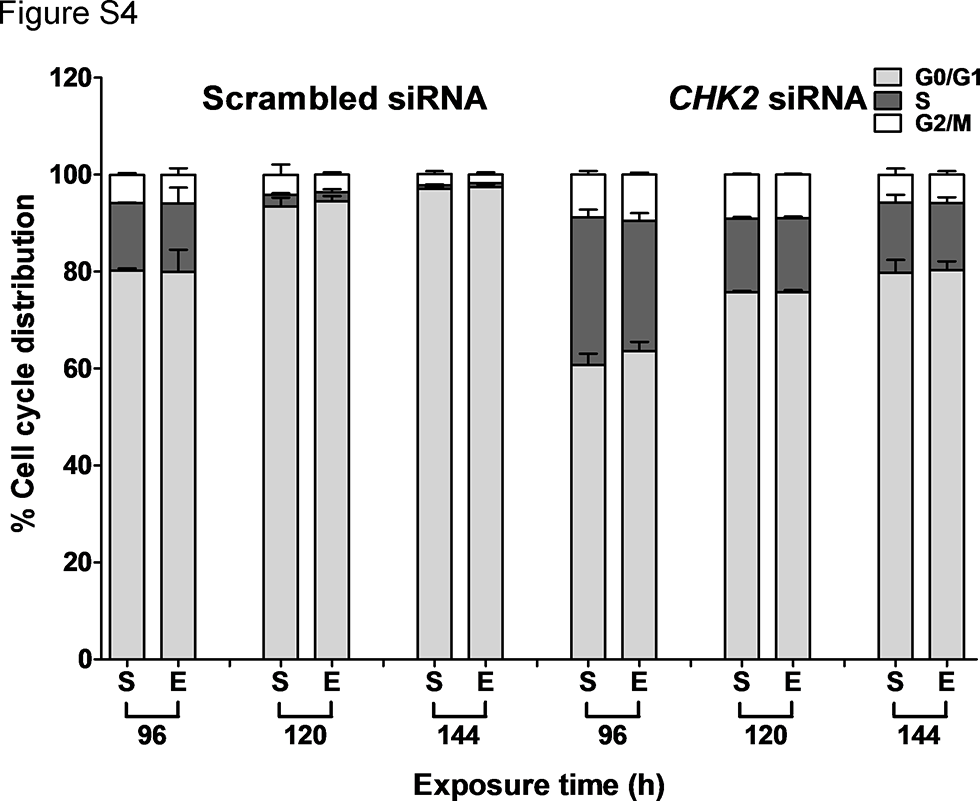

Supplement: Figure S4 — Cell cycle distribution in the sham (S) and exposed (E) of the CHK2 siRNA-treated and scrambled siRNA-treated HaCaT cells under ELF-EMF exposure. HaCaT cells were transfected with 5 nM of CHK2 siRNA or scrambled siRNA, followed by exposure to 1.5 mT ELF-EMFs for 96 to 144 h. After exposure to ELF-EMFs, the CHK2 siRNA-treated cells (right panel) and scrambled siRNA-treated cells (left panel) were analyzed by PI staining and flow cytometry. The percentage of the cell cycle growth phases are represented as the mean ± SD of three independent experiments. By applying a Student’s t-test on the data, there is no significant difference in cell cycle distribution between sham and exposed cells in CHK2 siRNA-treated (right panel) and scrambled siRNA-treated (left panel) groups respectively. (TIF) [file pone.0104732.s004.tif]
