# Supplementary material for: Extremely Low-Frequency Electromagnetic Fields Cause G1 Phase Arrest through the Activation of the ATM-Chk2-p21 Pathway
Source: PLoS One. 2014 Aug 11;9(8):e104732. doi: 10.1371/journal.pone.0104732 (PMC4128733; doi:10.1371/journal.pone.0104732)
Supplement: Table S1 — Number of differentially expressed genes (DEGs), and selected-genes (SGs; DEGs with at least 1.3 fold change) in each ELF-EMF-exposed group. (DOC) [file pone.0104732.s005.doc]

**Table S1. Numbers of differentially expressed genes (DEGs), and selected-genes (SGs; DEGs with at least 1.3 fold change) in each ELF-EMF-exposed group.**

| **Exposure Time** | 4 h | 8 h | 12 h | 24 h | 48 h | 72 h | 96 h | UV | Ci |
| --- | --- | --- | --- | --- | --- | --- | --- | --- | --- |
| **Numbers of DEGs** | 18 | 4 | 6 | 54 | 6 | 97 | 265 | 939 | 8 |
| **Numbers of SGs** | 1 | 1 | 1 | 4 | 2 | 28 | 102 | 614 | 0 |

4-96 h: exposure time of ELF-EMF; UV: positive control; Ci: sham exposure and internal control
