# Supplementary material for: Extremely Low-Frequency Electromagnetic Fields Cause G1 Phase Arrest through the Activation of the ATM-Chk2-p21 Pathway
Source: PLoS One. 2014 Aug 11;9(8):e104732. doi: 10.1371/journal.pone.0104732 (PMC4128733; doi:10.1371/journal.pone.0104732)
Supplement: Table S2 — The list of 102 selected genes (SGs) after 96 h of ELF-EMF exposure. (DOC) [file pone.0104732.s006.doc]

**Table S2. The list of 102 selected genes (SGs) after 96 h of ELF-EMF exposure.**

| **Gene Name** | **Entrez Gene ID** | **log_2_ ratio** |
| --- | --- | --- |
| GEM | 2669 | 0.95 |
| UCA1 | 652995 | 0.93 |
| CTSH | 1512 | 0.78 |
| OLFM4 | 10562 | 0.73 |
| CTSA | 5476 | 0.70 |
| ALPPL2 | 251 | 0.68 |
| S100A7 | 6278 | 0.64 |
| HIST1H1C | 3006 | 0.63 |
| CDA | 978 | 0.60 |
| SCNN1B | 6338 | 0.58 |
| ZNF358 | 140467 | 0.58 |
| SGPP1 | 81537 | 0.58 |
| PLA2G2A | 5320 | 0.56 |
| PSAP | 5660 | 0.55 |
| HIST1H2BD | 3017 | 0.55 |
| OAS1 | 4938 | 0.54 |
| CLDN1 | 9076 | 0.53 |
| CYP1B1 | 1545 | 0.53 |
| HIST1H2BK | 85236 | 0.53 |
| B4GALT4 | 8702 | 0.51 |
| GPX2 | 2877 | 0.49 |
| HIST2H2AA3 | 8337 | 0.48 |
| CDKN1A | 1026 | 0.48 |
| TSC22D3 | 1831 | 0.48 |
| IGFBP7 | 3490 | 0.47 |
| SEPT4 | 5414 | 0.47 |
| C17orf91 | 84981 | 0.44 |
| CD70 | 970 | 0.42 |
| CLU | 1191 | 0.42 |
| SC5DL | 6309 | 0.42 |
| CD3D | 915 | 0.40 |
| CROT | 54677 | 0.40 |
| ZKSCAN4 | 387032 | 0.40 |
| LZTS2 | 84445 | 0.40 |
| PARP6 | 56965 | 0.40 |
| TRIM29 | 23650 | 0.39 |
| TACSTD2 | 4070 | 0.39 |
| TP53 | 7157 | 0.39 |
| ISG15 | 9636 | 0.38 |
| SDCBP2 | 27111 | 0.38 |
| GNAI1 | 2770 | 0.38 |
| MYOZ3 | 91977 | -0.38 |
| ANXA1 | 301 | -0.39 |
| SH3D19 | 152503 | -0.40 |
| RPS24 | 6229 | -0.40 |
| SEPHS2 | 22928 | -0.41 |
| PSMD2 | 5708 | -0.41 |
| NMU | 10874 | -0.41 |
| ANXA3 | 306 | -0.41 |
| RPA3 | 6119 | -0.42 |
| RPS2 | 6187 | -0.44 |
| EBNA1BP2 | 10969 | -0.44 |
| C1QBP | 708 | -0.44 |
| RACGAP1 | 29127 | -0.46 |
| CAV1 | 857 | -0.46 |
| HSD17B10 | 3028 | -0.47 |
| AMPD2 | 271 | -0.47 |
| TSC22D1 | 8848 | -0.48 |
| KRT8 | 3856 | -0.49 |
| NME1 | 4830 | -0.49 |
| ARL6IP1 | 23204 | -0.51 |
| LGALS1 | 3956 | -0.51 |
| EFEMP1 | 2202 | -0.51 |
| SNRPA | 6626 | -0.52 |
| LOXL1 | 4016 | -0.52 |
| MTP18 | 51537 | -0.52 |
| CEP135 | 9662 | -0.52 |
| ENO1 | 2023 | -0.53 |
| RAN | 5901 | -0.59 |
| EMP1 | 2012 | -0.60 |
| SFRS3 | 6428 | -0.60 |
| MLLT10 | 8028 | -0.60 |
| ACTB | 60 | -0.64 |
| SNRPF | 6636 | -0.64 |
| BOLA3 | 388962 | -0.67 |
| CDC25B | 994 | -0.67 |
| ACTN2 | 88 | -0.69 |
| SERPINB7 | 8710 | -0.70 |
| KPNA2 | 3838 | -0.71 |
| TK1 | 7083 | -0.72 |
| ISYNA1 | 51477 | -0.75 |
| HSPA8 | 3312 | -0.75 |
| TUBB | 203068 | -0.78 |
| PTTG1 | 9232 | -0.80 |
| HMGB1 | 3146 | -0.83 |
| RANBP1 | 5902 | -0.85 |
| CDC2 | 983 | -0.86 |
| BIRC5 | 332 | -0.86 |
| TSEN15 | 116461 | -0.89 |
| OIP5 | 11339 | -0.91 |
| CCNA2 | 890 | -0.93 |
| CDC20 | 991 | -0.95 |
| KIF23 | 9493 | -0.96 |
| KIAA0101 | 9768 | -0.96 |
| KIF20A | 10112 | -1.03 |
| HMMR | 3161 | -1.05 |
| CCNB1 | 891 | -1.11 |
| UBE2C | 11065 | -1.23 |
| TUBA1B | 10376 | -1.24 |
| TUBA1C | 84790 | -1.27 |
| FOS | 2353 | -1.34 |
| TLR4 | 7099 | -1.52 |
